# Loneliness in Young Adults During the First Wave of COVID-19 Lockdown: Results From the Multicentric COMET Study

**DOI:** 10.3389/fpsyt.2021.788139

**Published:** 2021-12-10

**Authors:** Gaia Sampogna, Vincenzo Giallonardo, Valeria Del Vecchio, Mario Luciano, Umberto Albert, Claudia Carmassi, Giuseppe Carrà, Francesca Cirulli, Bernardo Dell'Osso, Giulia Menculini, Martino Belvederi Murri, Maurizio Pompili, Gabriele Sani, Umberto Volpe, Valeria Bianchini, Andrea Fiorillo

**Affiliations:** ^1^Department of Psychiatry, University of Campania Luigi Vanvitelli, Naples, Italy; ^2^Department of Medicine, Surgery and Health Sciences, University of Trieste, Trieste, Italy; ^3^Department of Mental Health, Azienda Sanitaria Universitaria Giuliano Isontina—ASUGI, Trieste, Italy; ^4^Department of Clinical and Experimental Medicine, University of Pisa, Pisa, Italy; ^5^Department of Medicine and Surgery, University of Milan Bicocca, Milan, Italy; ^6^Center for Behavioral Sciences and Mental Health, National Institute of Health, Rome, Italy; ^7^Department of Biomedical and Clinical Sciences, Luigi Sacco and Aldo Ravelli Center for Neurotechnology and Brain Therapeutic, University of Milan, Milan, Italy; ^8^Department of Psychiatry, University of Perugia, Perugia, Italy; ^9^Department of Biomedical and Specialty Surgical Sciences, Institute of Psychiatry, University of Ferrara, Ferrara, Italy; ^10^Department of Neurosciences, Mental Health and Sensory Organs, Faculty of Medicine and Psychology, Sapienza University of Rome, Rome, Italy; ^11^Department of Neuroscience, Section of Psychiatry, University Cattolica del Sacro Cuore, Rome, Italy; ^12^Department of Psychiatry, Fondazione Policlinico Agostino Gemelli IRCCS, Rome, Italy; ^13^Clinical Psychiatry Unit, Department of Clinical Neurosciences, Università Politecnica delle Marche, Ancona, Italy; ^14^Department of Life, Health and Environmental Sciences, Psychiatric Unit: Trattamenti Riabilitativi Psicosociali, Interventi Precoci, TRIP, Psychosocial Rehabilitation Treatment, Early Interventions University Unit, University of L'Aquila, L'Aquila, Italy

**Keywords:** loneliness, trauma, pandemic, mental disorders, youth

## Abstract

The COVID-19 pandemic has affected the mental and physical health of the general population at any age, but it is expected to have a protracted and severe consequences for younger populations. The pandemic has had several consequences on mental health including anger and irritability, depressive symptoms and somatic complaints, insomnia, lack of motivation, and loneliness. In particular, loneliness and its related negative feelings are thought to be particularly pronounced during young adulthood because of the many social changes that young people deal with during this period of life. Therefore, it is essential to evaluate the type of impact of the pandemic on the mental health of young people and their levels of loneliness experienced during the first phase of the lockdown. Based on the largest Italian study on the effects of the COVID-19 pandemic on the mental health of general population, in this paper we aim to: (1) describe the levels of loneliness in a national sample of Italian young adults aged 18–34 years, during the first wave of lockdown in 2020; (2) evaluate the clinical and socio-demographic differences in young adults reporting low vs. high levels of loneliness; (3) assess the role of clinical symptomatology, coping strategies, levels of resilience, and duration of lockdown as possible predictors of loneliness. The final sample consists of 8,584 people, mainly female (72.6%), single, with a mean age of 26.4 (±4.4) years. The mean score at the UCLA was 47.5 (±13.6), with 27% (*N* = 2,311) of respondents exceeding the cut-off for high levels of loneliness. High levels of loneliness were predicted by the presence of avoidant coping strategies, such as self-distraction (Beta coefficient, B = 0.369, 95% Confidence Interval, CI = 0.328–0.411), venting (B = 0.245, 95% CI = 0.197–0.293), denial (B = 0.110, 95% CI = 0.061–0.159), and emotional disengagement (B = 0.133, 95% CI = 0.080–0.185). Weeks of exposure to the pandemic were significantly associated with worsening of loneliness (*p* < 0.000). There is currently considerable interest in trying to reduce loneliness, both within the context of COVID-19 and more generally. Our results highlight that young people are at a higher risk of developing loneliness and suggest that more interventions and practical guidelines are needed.

## Introduction

The COVID-19 pandemic is a “new” form of trauma affecting different groups of individuals, communities, cities, regions at the same time, with no possibility to identify and limit the “enemy” (1–3). Although the pandemic has affected the mental and physical health of the general population at any age (4–6), it is expected that this global crisis can have protracted and severe consequences for younger populations (7). In fact, the pandemic is posing multiple challenges to young people, through the disruption of daily educational, academic, professional, social, and family life (8, 9).

Due to the rapid spread of the coronavirus, several preventive measures, including physical distance, face masks, home quarantine, and lockdown restrictions have been implemented in order to contain the transmission and the contagion of other people (10, 11).

On March 8, 2020, the Italian Prime Minister announced the “stay at home” order and entered the first national lockdown in response to the COVID-19 pandemic. Based on previous experience with infectious disease outbreaks, quarantine, lockdown, and physical distancing are unpleasant experiences, involving separation from loved ones, uncertainty, and unemployment (12), increased mental distress and post-traumatic symptoms (13–15), anger and irritability (11, 16), depressive symptoms and somatic complaints (17, 18), insomnia (19, 20), suicidal ideation (21, 22), lack of motivation and loneliness (11, 16, 23, 24). In the general population, the adoption of different coping styles (25), the levels of resilience (26) and familiarity with mental disorders (27) have been identified as the most relevant moderators of the impact of the pandemic on mental health (2).

Loneliness is defined as a negative emotion related with the discrepancy between desired and existing relations, and it can be either emotional and social (28, 29). Emotional loneliness is described as a subjective experience resulting from the absence of a close bonding with a person, whereas social loneliness reflects an objective lack of contacts and social networks (30, 31).

Loneliness represents a major public health concern, since it is associated with an increased risk of depressive disorders, anxiety disorders, and suicidal ideation (32), as well as of cardiovascular disease, stroke, coronary heart disease (33, 34), cognitive decline (35), and increased all-cause mortality risk (36–38).

Loneliness and its related negative feelings are thought to be particularly pronounced during adolescence and young adulthood (39) because of the many social and personal changes that young people deal with during this period of life (40–43). Loneliness itself has been referred to as an epidemic, and there have been heightened concerns about its effects during the COVID-19 pandemic (8, 44–46). Therefore, it is essential to evaluate the type of impact of the pandemic and the levels of loneliness experienced by young people (47). By disentangling such complex relationship between pandemic and loneliness, it would be possible to develop *ad-hoc* preventive strategies targeting the young people (48–50), which are expected to be the most severely hit by the long-term consequences of the pandemic (51–53).

Based on the largest Italian study on the effects of the COVID-19 pandemic on the mental health of general population (27), in this paper we aim to: (1) describe the levels of loneliness in a national sample of Italian young adults aged 18–34 years, during the first wave of lockdown in 2020; (2) evaluate the clinical and socio-demographic differences in young adults reporting low vs. high levels of loneliness; (3) assess the role of clinical symptomatology, coping strategies, levels of resilience, and duration of lockdown as possible predictors of loneliness.

## Materials and Methods

The COvid Mental hEalth Trial (COMET) is a national trial coordinated by the University of Campania “Luigi Vanvitelli” (Naples) in collaboration with nine university sites: Università Politecnica delle Marche (Ancona), University of Ferrara, University of Milan Bicocca, University of Milan “Statale,” University of Perugia, University of Pisa, Sapienza University of Rome, “Catholic” University of Rome, University of Trieste. The Center for Behavioral Sciences and Mental Health of the National Institute of Health in Rome has been involved in the study by supporting the dissemination and implementation of the project according to the clinical guidelines produced by the National Institute of Health for managing the effects of the COVID-19 pandemic. The COMET was conceived as a cross-sectional observational design using a snowball sampling method for the recruitment of the Italian general population. The full study protocol is available elsewhere (54).

The main outcome measure of the study is the DASS-21, evaluating the general distress on a tripartite model of psychopathology (55, 56). It consists of 21 items grouped in three subscales: depression, anxiety and stress.

The levels of loneliness have been evaluated by the UCLA scale short version, which includes 10 items rated on a 4-level Likert scale (57). Higher values indicate higher levels of loneliness. As reported by Morahan-Martin and Schumacher (58), participants in the highest 20% of responses were classified as “Lonely,” compared with all other participants (“Non-lonely”).

Respondents' socio-demographic (e.g., gender, age, geographical region, working, and housing condition, etc.) and clinical information (e.g., having a previous physical or mental disorder, using illicit drugs or medications, etc.) have been collected through *ad-hoc* schedules. Other validated and reliable questionnaires included in the study are: the General Health Questionnaire-12 items version (GHQ) (59); the Obsessive-Compulsive Inventory—Revised version (OCI-R) (60), the Insomnia Severity Index (ISI) (61), the Suicidal Ideation Attributes Scale (SIDAS) (62), the Severity-of-Acute-Stress-Symptoms-Adult scale (SASS) (63), the Impact of Event Scale—short version (IES) (64), the Connor-Davidson resilience scale (65), the brief-COPE (66), the short form of Post-Traumatic Growth Inventory (PTGI) (67), the Multidimensional Scale of Perceived Social Support (MSPPS) (68), and (only for healthcare professionals) the Maslach Burnout Inventory (MBI) (69). The present paper is based on a sub-analysis of the sample of young people. A previous survey promoted by the International Labor Organization in the UK, entitled “Youth and COVID-19,” has selected participants aged 18–34 years and therefore the same age group has been considered in the present paper (70).

### Ethics and Dissemination

This study is being conducted in accordance with globally accepted standards of good practice, in agreement with the Declaration of Helsinki and with local regulations.

The participants provided their written informed consent to participate in this study. The study has been approved by the Ethical Review Board of the University of Campania “L. Vanvitellii” (Protocol number:0007593/i).

### Statistical Analysis

Descriptive statistics were performed in order to describe the socio-demographic and clinical characteristics of the sample. *T*-test for independent samples and Chi-square have been performed to test differences between “lonely” vs. “non-lonely” participants, as appropriate.

In order to identify possible predictors of the levels of loneliness, a multivariate linear regression model, weighted for the propensity score, was performed, including as independent variables: adaptive and maladaptive coping strategies, having been infected by the COVID-19, having a pre-existing mental disorder, being a healthcare professional. Furthermore, in order to evaluate the impact of the duration of lockdown and of other containment measures on the primary outcomes, the categorical variable “Week” was also entered in the regression models. The models were adjusted for the rate of new COVID-19 cases and of COVID-related mortality during the study period, as well as for several socio-demographic characteristics, such as gender, age, occupational status, having a physical comorbid condition, hours spent on Internet, health status, number of cohabiting people, satisfaction with one's own life, with cohabiting people, and with housing condition. All variables have been managed as reported in detail elsewhere (27).

Missing data have been handled using the multiple imputation approach. Statistical analyses were performed using the Statistical Package for Social Sciences (SPSS), version 17.0 and STATA, version 15. For all analyses, the level of statistical significance was set at *p* < 0.05.

## Results

### Global Sample

The final sample consists of 8,584 people, mainly female (72.6%, *N* = 6,232), single, with a mean age of 26.4 (±4.4). 58.2% of them (*N* = 5,000) have a university degree and 71.4% (*N* = 6,131) are single; 7.1% of respondents have lost their job or interrupted their studies during the pandemic.

The majority of participants reported to enjoy their living conditions (66.8%, *N* = 5,732) and to live with their co-habitants (70.5%, *N* = 6,055), while 24% of participants (*N* = 2,094) reported not to be satisfied with their own life. 5.4% (*N* = 461) participants reported to suffer from a mental disorder and 6.7% (*N* = 573) of a physical disorder.

**Figure 1:**
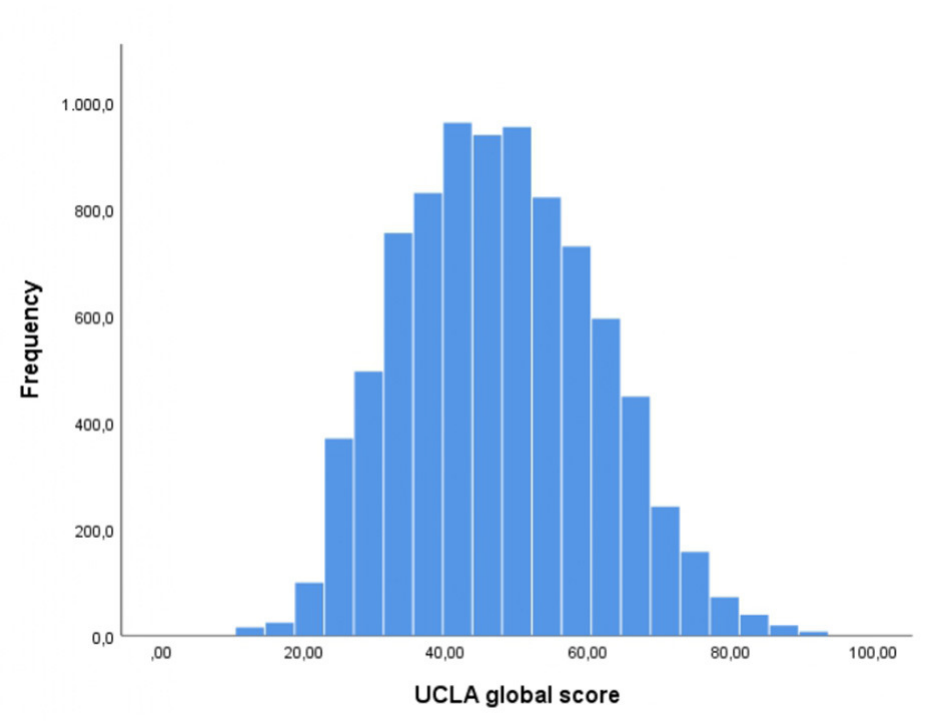
Distribution of the levels of loneliness.

The mean score at the UCLA was 47.5 (±13.6), with 27% (*N* = 2,311) of respondents exceeding the cut-off for high levels of loneliness (Table 1, Figure 1).

**Table 1:** Socio-demographic characteristics of the sample (*N* = 8,584).

	**Global sample (*N =* 8,584)**	**Lonely (*N =* 2,311)**	**Not-lonely (*N =* 6,273)**	***P*-value**
Gender, female, % (*N*)	72.6 (6,232)	73.2 (1,691)	72.4 (4,541)	0.471
Age, M (SD)	26.4 (±4.4)	26.2 (4.4)	26.5 (4.4)	0.002
**Age category**				
18–29 ys	70.9 (6,088)	73 (1,686)	70.2 (4,402)	0.012
30–34 ys	29.1 (2,496)	27 (625)	29.8 (1,871)	
Marital status, single, yes, % (*N*)	71.4 (6,131)	72.3 (1,671)	71.1 (4,460)	0.346
Student, yes, % (*N*)	51.6 (4,429)	52.5 (3,293)	51 (3,201)	
Employed, yes, % (*N*)	51.6 (4,429)	52.5 (3,293)	49.2 (1,136)	
Lost job/interrupted educational activities, yes, % (*N*)	7.1 (606)	7.0 (162)	7.1 (444)	0.913
Any physical disorder, yes, % (*N*)	6.7 (573)	7.1 (165)	6.5 (408)	0.295
Any mental disorder, yes, % (*N*)	5.4 (461)	5.5 (126)	5.3 (335)	0.829
Educational level, university, yes, % (*N*)	58.2 (5,000)	55.2 (1,276)	59.4 (3,724)	0.013
Being infected by COVID, yes, % (*N*)	5.3 (453)	4,7 (109)	5,5 (344)	0.174
Severely hit region, yes, % (*N*)	28.6 (2,452)	28.2 (651)	28.7 (1,801)	0.628

### Differences in “Lonely” vs. “Non-lonely” Respondents

Participants from the “lonely” group reported a higher severity of depressive, anxiety, and stress-related symptoms (DASS-Depression: 13.8 ± 6.8; DASS-anxiety: 8.7 ± 7.0; DASS-stress:17.2 ± 6.1), compared to the “non-lonely” sample (*p* < 0.0001) (Table 2).

**Table 2:** Differences in clinical features between lonely and not-lonely participants.

	**Global sample (*****N** **=*** **8,584)**		**Not-lonely (*****N** **=*** **6,273)**		**Lonely (*****N** **=*** **2,311)**		***P*-value**
	***M***	***SD***	***M***	***SD***	***M***	***SD***	
DASS stress	16.9	6.3	16.8	6.4	17.2	6.1	0.024
DASS anxiety	8.4	6.9	8.3	6.9	8.7	7.0	0.006
DASS depression	13.4	7.0	13.3	7.1	13.8	6.8	0.005
GHQ global score	17.4	3.3	17.3	3.2	17.4	3.2	0.288
OCI global score	12.0	8.7	11.8	8.7	12.5	8.7	0.001
SASS global score	7.1	5.3	7.0	5.3	7.5	5.4	0.001
Connor global score	31.3	10.4	32.5	10.2	27.9	10.3	0.000
ISI global score	7.2	5.4	7.1	5.4	7.4	5.3	0.517
IES intrusive	2.1	1.9	2.1	1.9	2.1	1.9	0.309
IES avoidance	2.4	1.9	2.4	1.9	2.3	1.9	0.084
IES hyperarousal	2.5	1.9	2.5	1.9	2.5	1.9	0.674
PTGI—relating to others	1.9	1.4	1.9	1.4	1.9	1.4	0.014
PTGI—new possibilities	1.8	1.3	1.9	1.3	1.7	1.3	0.000
PTGI—personal strenght	2.2	1.5	2.2	1.5	2.1	1.5	0.000
PTGI—spiritual help	1.2	1.2	1.2	1.3	1.2	1.2	0.249
PTGI—appreciation life	2.3	1.4	2.4	1.4	2.3	1.4	0.000
Support—family	21.1	6.8	21.9	6.4	19.0	7.3	0.000
Support—friends	20.4	6.6	21.1	6.3	18.7	7.0	0.000
Support—others	22.4	6.7	23.0	6.3	20.7	7.4	0.000
SIDAS global score	4.8	6.6	4.7	6.5	5.1	7.1	0.000
Suicidal ideation, yes, % (*N*)	14.2 (1,216)		14.3 (898)		13.8 (318)		0.534
Above OCI threshold, yes, % (*N*)	14.6 (1,249)		14.1 (884)		15.8 (365)		0.045

Moreover, 15.8% (*N* = 365) of the “lonely” sample scored above the threshold for clinical relevance of obsessive-compulsive symptomatology, with a global severity of obsessive–compulsive symptoms of 12.5 ± 8.7 at OCI-R, significantly higher than the “non-lonely” group (*p* < 0.05). Suicidal ideation was reported by 13.8% (*N* =318) of the “lonely” group, with a mean score of 5.1 ± 7.1 at the SIDAS, compared to the 14.3% in the “not-lonely group” (4.7 ± 6.5).

The levels of resilience were significantly lower in participants from the “lonely” group, compared with the remaining sample (*p* < 0.0001).

People from the “lonely” group also reported to use maladaptive coping strategies very frequently. In particular, self-distraction was frequently used in 38% of cases (vs. 24% of cases in the “non-lonely” group, *p* < 0.0001), self-blame in 25% of cases (vs. 14.4%, *p* < 0.0001) and venting in 15.4% (vs. 8.4%, *p* < 0.0001). On the contrary, people from the “non-lonely” group more frequently used adaptive coping strategies, such as acceptance (47.7% of cases vs. 35.1%; *p* < 0.0001), planning (40.3 vs. 32%; *p* < 0.0001), and positive reframing (29% vs. 21.2%; *p* < 0.0001).

### Predictors of Loneliness

According to the multivariate regression model, weighted for the propensity score, high levels of loneliness were predicted by the presence of avoidant coping strategies, such as self-distraction (Beta coefficient, B = 0.369, 95% Confidence Interval, 95% CI = 0.328–0.411), venting (B = 0.245, 95% CI = 0.197–0.293), denial (B = 0.110, 95% CI = 0.061–0.159), and emotional disengagement (B = 0.133, 95% CI = 0.080–0.185). Interestingly, the levels of loneliness were reduced by using adaptive coping strategies, such as search for information (B = −0.125, 95% CI = −0.184 to −0.066), planning (B = −0.106, 95% CI = −0.159 to −0.053) and positive reframing (B = −0.080, 95% CI= −0.127 to −0.034).

Weeks of exposure to the pandemic and to the related containment measures were significantly associated with worsening of loneliness, with Beta coefficient ranging from B = 0.4 (95% CI: 0.078–0.830) during the week April 16–22 to B = 0.323 (95% CI: 0.112–0.534) in the week April 30–May 4 (*p* < 0.000).

Being infected by COVID-19 and having a pre-existing mental or physical disorder did not impact on the levels of perceived loneliness, even after controlling for gender, age, living in the most severely affected areas, infection rate, and mortality rate for COVID-19 in Italy. We also found that the severity of depressive, anxiety or stress symptoms, of obsessive-compulsive symptoms, of insomnia, post-traumatic symptoms, and suicidal ideation did not have any influence on the levels of loneliness (Table 3).

**Table 3:** Predictors of the levels of loneliness.

	**B**	**Sign**.	**95% Confidence Interval**	
			**Lower bound**	**Upper bound**
Intercept	10,367	0.000	9,310	11,424
Being infected by COVID, yes	0.105	0.525	−0.220	0.431
Gender, female ref.	0.096	0.554	−0.223	0.416
Severely hit area, yes	0.097	0.173	−0.042	0.236
Pre-existing physical disorder, yes	−0.076	0.272	−0.210	0.059
Pre-existing mental disorder, yes	−0.106	0.393	−0.351	0.138
**Time to exposure, ref. week March 30—April 8**				
Week April 15–April 9	0.265	0.299	−0.235	0.765
Week April 16–April 22	0.454	0.018	0.078	0.830
Week April 23–April 29	0.198	0.246	−0.137	0.533
Week April 30–May 4	0.323	0.003	0.112	0.534
Quarantine	−0.211	0.043	−0.415	−0.006
Cases COVID	3,775	0.586	−9,830	0.000
Death COVID	0.001	0.157	0.000	0.002
Age, ref. cat. 18–29 ys (vs. 30–34 ys)	0.057	0.415	−0.079	0.192
Being student, yes	0.073	0.372	−0.087	0.232
Being employed, yes	−0.162	0.044	−0.004	−0.320
DASS anxiety	−0.002	0.695	−0.013	0.009
DASS depression	0.004	0.406	−0.006	0.015
DASS stress	0.002	0.689	−0.009	0.013
GHQ global score	−0.005	0.644	−0.024	0.015
OCI global score	0.007	0.121	−0.002	0.017
ISI global score	0.007	0.315	−0.006	0.020
IES global score	0.002	0.770	−0.010	0.014
SASS global score	−0.006	0.504	−0.022	0.011
Support from others	−0.038	0.000	−0.050	−0.027
Support from friends	−0.038	0.000	−0.050	−0.026
Support from family	−0.049	0.000	−0.060	0.038
Resilience levels	−0.034	0.000	−0.042	−0.027
COPE—Self-distraction	0.369	0.000	0.328	0.411
COPE- Active	−0.026	0.311	−0.075	0.024
COPE—Denial	0.110	0.000	0.061	0.159
COPE—substance use	0.121	0.000	0.065	0.177
COPE—emotional support	0.418	0.000	0.361	0.475
COPE—information	−0.125	0.000	−0.184	−0.066
COPE emotional Disengagement	0.133	0.000	0.080	0.185
COPE—venting	0.245	0.000	0.197	0.293
COPE—positive reframing	−0.080	0.001	−0.127	−0.034
COPE—planning	−0.106	0.000	−0.159	−0.053
COPE—humor	0.020	0.380	−0.024	0.064
COPE—acceptance	−0.036	0.157	−0.086	0.014
COPE—religion	−0.077	0.000	−0.116	−0.037
PTGI—relating to others	0.017	0.595	−0.047	0.082
PTGI—new possibilities	0.059	0.155	−0.022	0.140
PTG—personal strength	−0.065	0.063	−0.133	0.004
PTGI—spiritual help	−0.012	0.764	−0.093	0.068
PTGI—appreciation life	0.160	0.000	0.102	0.219

Finally, high levels of post-traumatic growth, such as appreciation for life (B = 0.160, 95% CI = 0.102–0.219) were a significant protective factor for levels of loneliness (Table 3).

## Discussion

The COVID-19 pandemic represents an unprecedent traumatic event, which has completely disrupted the daily routine of the general population worldwide for more than a year now (71). The mental health of young adults has not been fully considered during the first weeks of the pandemic, although it was clear already from the beginning that young people would have been a group at higher risk of developing long-term physical, mental and social problems (72). In particular, the enforced physical isolation due to the public health containment measures can be associated with a subjective feeling of loneliness, which represent a specific dimension to be carefully monitored for the prevention of mental health problems (73). In many individuals, especially the younger ones, the lockdown and physical distancing can have increased the perception of social isolation. Although social isolation—defined as the absence of social interactions, contacts and relationships with others—is conceptually distinguished from loneliness—that is the feeling that one's social needs are not being met by the quantity or quality of one's social relationships, these two dimensions appear to be strongly interrelated, with physical and social isolation being a risk factor for becoming “lonely” (74).

During the ongoing health crisis, there have been calls to ascertain how the pandemic has affected loneliness to ensure that at-risk individuals receive all the necessary support. Therefore, in our study we decided to describe the levels of loneliness in a national sample of Italian adults, during the first wave of the lockdown in 2020, as one of the considered outcome measures.

In the sub-sample of young people aged 18–34 years, the levels of loneliness were quite high, being particularly severe in a third of cases. This is an expected finding, in line with those from other studies carried out in Europe, which highlighted that younger adults, women, people with low income, and those with mental health problems are more likely to be in the highest loneliness class relative to the lowest (75, 76). According to the COVID-19 Psychological Well-being study, people of younger age reported more severe levels of loneliness and were four to five times more likely to report loneliness, compared with older adults (77). In a sample of US young adults aged 18–35 years, the prevalence of loneliness was estimated at 43% (78). In this sample, results indicated a trend toward moderate levels of loneliness, with women reporting significantly more feelings of loneliness compared to men. In our sample, we did not find gender differences in the levels of loneliness, but this finding could be due to the overrepresentation of female population compared to male.

Furthermore, young people in the highest loneliness cluster reported a higher severity of depressive, anxiety and stress symptoms, of obsessive-compulsive symptomatology, and a high rate of suicidal ideation, even compared with the global COMET sample (27). As expected, people belonging to the group with highest rates of loneliness were those with more severe levels of psychiatric symptoms, confirming that loneliness is strongly associated with depression and other mental disorders both in old (79) and young adults (80, 81). Although loneliness has been traditionally linked with older adults (32, 82, 83), even young adults up to the age of 25 may experience high levels of loneliness (55, 84). These data confirm the hypothesis that loneliness can represent a useful dimension to be carefully monitored in routine clinical practice by healthcare professionals working with adolescents and young people (85, 86).

Another interesting finding is that adaptive coping strategies, such as planning and positive reframing, work as protective factors against loneliness. This is a relevant finding considering that coping strategies may easily change following specific psychosocial interventions, such as psychoeducation (87, 88) and problem-solving oriented interventions (89) or by improving the levels of resilience (25, 81, 90, 91).

Furthermore, we also found that the levels of loneliness tend to increase over time, being more severe in the last weeks of the lockdown, as confirmed in our regression models controlled for all respondents' socio-demographic characteristics. This finding confirms the hypothesis that the duration of containment measures significantly influences mental health and well-being of the general population (27, 92, 93). The same trend in the levels of self-reported loneliness was reported by Bu et al. (76), who described a U-shaped trajectory in the levels of loneliness in the period June-November 2020, corresponding to the limitations to social activities due to the lockdown policies (94). In fact, young people aged between 18 and 29 reported higher levels of loneliness, but with a decrease in loneliness in the first period of the pandemic, from March to May 2020 (76, 95–97).

In addition, being employed and being a student were associated with a lower risk of loneliness during the pandemic. This is consistent with Arnett's theory (98) that working and education status might potentially be signs of age-specific personal achievements (99). Moreover, school and workplace may also help young adults to increase their social network and to reduce social disconnection (100–102). Prolonged school closures, strict social isolation from peers, extended family, and community networks, economic shutdown, and the pandemic itself may have contributed to the mental health problems of many adolescents and young adults (103–105). Being confined to home leads to disturbances in sleep/wake cycles and physical exercise routines, and promotes the excessive use of technology (106–109). Further studies are needed in order to evaluate the long-term effects of these conditions on the development of full-blown mental health disorders (110, 111).

The present study has some limitations which are hereby acknowledged. First, the snowball sampling methodology could have led to a selection bias, with only those interested in the psychological consequences of the pandemic willing to participate (112). Second, the cross-sectional design of the survey prevents us to delineate any causal relationship between the selected variables. Third, several variables which could have had an impact on the levels of perceived loneliness, such number of contacts with peers (prior and during the pandemic), time spent with friends/peer, quality of social relationship, number of social activities in which they are usually involved, desire for social contact (113–115), the quality of family communication styles, and the individual acceptance and attitudes toward restrictive measures related to the pandemic, have not been collected in our study. Fourth, it is very difficult to disentangle the complex relationship between the exposure to COVID-19 pandemic and other contextual factors contributing to the levels of loneliness. In this study, the proxy measure selected is represented by the variable “weeks of lockdown,” but it should be acknowledged that many other variables could have had an influence on the loneliness levels.

Finally, our sample cannot be considered fully generalizable of all young people because we could recruit only people aged 18 years or more.

Among its strengths, we should consider that this is one largest surveys carried out in Italy on the effects of the COVID-19 pandemic on the mental health and well-being of the general population. Moreover, given the large sample size, we could analyze differences and similarities between young and old people, but also between males and females (116). Finally, the large battery of used tests allowed us to test the effects of loneliness on several mental health dimensions and symptoms.

Overall, our findings suggest that the levels of loneliness during the weeks of the first strict lockdown were high in young adults. People using maladaptive coping strategies, such as self-blame or self-distraction, were at higher risk for reporting highest levels of loneliness. However, certain social factors such as having close friends, having strong perceived social support, having high levels of resilience and using adaptive coping strategies were protective factors. There is currently considerable interest in trying to reduce loneliness within society, both within the context of COVID-19 pandemic and more generally (117–121). Our results highlight that young people are at a higher risk of developing loneliness and suggest that more interventions and practical guidelines are needed.

## Data Availability Statement

The data presented in this study are available on request from the corresponding author.

## Ethics Statement

The studies involving human participants were reviewed and approved by Ethical Committee of the University of Campania L. Vanvitelli. The participants provided their written informed consent to participate in this study.

## Author Contributions

VG, GSam, and AF: conceptualization. ML and VV: methodology. GS: formal analysis. UA, CC, GSan, GC, MP, UV, BD, MBM, and FC: investigation. GS, VB, and GM: writing—original draft preparation. AF, UA, CC, GSan, MP, UV, BD, MBM, and FC: writing—review and editing. All authors have read and agreed to the published version of the manuscript.

## Conflict of Interest

The authors declare that the research was conducted in the absence of any commercial or financial relationships that could be construed as a potential conflict of interest. The handling editor declared a shared affiliation with one of the authors UV at the time of review.

## Publisher's Note

All claims expressed in this article are solely those of the authors and do not necessarily represent those of their affiliated organizations, or those of the publisher, the editors and the reviewers. Any product that may be evaluated in this article, or claim that may be made by its manufacturer, is not guaranteed or endorsed by the publisher.
